# Monodansylpentane as a Blue-Fluorescent Lipid-Droplet Marker for Multi-Color Live-Cell Imaging

**DOI:** 10.1371/journal.pone.0032693

**Published:** 2012-03-01

**Authors:** Huei-Jiun Yang, Chia-Ling Hsu, Jin-Yi Yang, Wei Yuan Yang

**Affiliations:** 1 Institute of Biological Chemistry, Academia Sinica, National Taiwan University, Taipei, Taiwan; 2 Institute of Biochemical Sciences, College of Life Sciences, National Taiwan University, Taipei, Taiwan; Institute of Molecular and Cell Biology, Singapore

## Abstract

Lipid droplets (LDs) are dynamic cellular organelles responsible for the storage of neutral lipids, and are associated with a multitude of metabolic syndromes. Here we report monodansylpentane (MDH) as a high contrast blue-fluorescent marker for LDs. The unique spectral properties make MDH easily combinable with other green and red fluorescent reporters for multicolor fluorescence imaging. MDH staining does not apparently affect LD trafficking, and the dye is extraordinarily photo-stable. Taken together MDH represents a reliable tool to use for the investigation of dynamic LD regulation within living cells using fluorescence microscopy.

## Introduction

Lipid droplets (LDs) are ubiquitous cellular organelles responsible for the storage of neutral lipids, important in development, and provide needed energy supply under conditions where food sources become limiting [1], [2]. Notable associations to LD dysregulation are a multitude of metabolic syndromes, such as obesity and diabetes. LDs are dynamic: proteomic analysis has revealed that large numbers of proteins reside on LD surfaces, with the exact protein content dynamically remodeled under varying culturing conditions [3], [4], [5], [6]. LDs are actively transported along microtubules, oftentimes undergoing fusion events [7], [8], [9], [10]. Live cell analysis is thus required for unraveling the dynamic regulation of this important organelle.

Live-cell imaging offers a unique opportunity for investigating LD regulation. A large collection of imaging tools based on fluorescence is currently available to cell biologists. Varying colors of photostable genetically-encoded fluorescent proteins can be used in multiplexed tracking of protein remodeling on LDs [11]. Photo-switchable fluorescent proteins as well as fluorescent timers will allow quantitative assessment of LD protein dynamics [12], [13]. Fluorescent sensors, such as those based on fluorescence resonance energy transfer (FRET), can be applied to follow protein conformational changes or protein-protein interactions relevant to LDs [14]. These tools, combined with the use of a reliable LD marker, represent a versatile scheme for investigating LD biology.

However, commercially available live-cell LD dyes such as NileRed and BODIPY 493/503 can be limiting in multicolor imaging, as most ready-made fluorescent reporter constructs fluoresce in the green to red region of the visible spectrum (e.g. the large collection of reporter constructs available from Addgene utilizing EGFP, EYFP or mcherry). NileRed emits broadly between green and red, while its absorption significantly overlaps with both EGFP and mcherry, making combined use with other fluorescent reporters difficult. BODIPY 493/503, on the other hand, can be used together with red-fluorescent constructs for two-color imaging; but its lesser photo stability, and being photoconvertable from green to red fluorescent [15], limit one's ability to utilize it for time lapse imaging of living cells. Two recently reported, but commercially unavailable small molecules, LD540 and LipidGreen, were demonstrated as probes for *in vivo* imaging of lipid droplets [16], [17], but again fluoresce in the green to red region of the visible spectrum. Dye-free LD imaging can be achieved through coherent Raman microscopy [18], [19] or harmonic generation microscopy [20], [21], though these methods require sophisticated setups that are less accessible. Combining these dye-independent methodologies with fluorescence in live-cell imaging could also be problematic, as pulsed-lasers required for these techniques rapidly photobleach fluorescent proteins, rendering longer-term investigations difficult. The identification of a dye that can stain the LD cores while being spectrally well-separated from most fluorescence reporters will be key to the application of live-cell imaging in studying LD biochemistry.

Herein we report a commercially available fluorophore monodansylpentane (MDH) as a new marker for LDs in living cells. MDH absorbs in the violet (and emits blue fluorescence when labeling LDs), well-separated from the most robust fluorescent proteins/reporters. It can be excited with 405 nm, compatible with most confocal fluorescence microscopes. We found MDH achieved its high contrast LD labeling in living cells through two mechanisms: its preferential partitioning into LDs, and its significantly blue-shifted, highly enhanced emission within lipophilic environments. Easily combinable with different fluorescent reporters for multi-color live cell imaging, MDH possess extraordinary photostability as compared to NileRed and BODIPY 493/503. MDH can also be effectively imaged with two-photon fluorescence microscopy. MDH labeling did not apparently affect LD trafficking activities in living cells. Taken together, these findings demonstrate that MDH represents a robust marker for LDs in live-cell fluorescence microscopy.

## Materials and Methods

### Materials

Methanol (#1060074000) was obtained from Merck. 2-propanol (#9084-03) was obtained from J.T. Baker. 100% sunflower seed oil was obtained from Quaker (Taiwan). BODIPY 493/503 (#D-3922) was obtained from Invitrogen. The EGFP-tubulin plasmid (#6117-1) was obtained Clonetech. The TagRFP-ADRP plasmid was constructed by inserting the human ADRP gene into the pTagRFP-C vector (#FP-141) from Evrogen. The mEos2-GABARAPL2 plasmid was constructed by combining mEos2 (Addgene, #20341) and the human GABARAPL2 gene in the pEGFP-C1 vector backbone (Clonetech).

### Cell culture and transfection

HeLa cells (CCL-2) were acquired from American Type Culture Collection (ATCC, Manassas, VA, USA) and maintained at 37°C, 5*%* CO_2_ in Dulbecco's Modified Eagle Medium (Gibco, #11965) supplemented with 10% Fetal Bovine Serum (Gibco, #10437) and 1% P/S (Gibco, #15140). HepG2 cells (HB-8065) were acquired from American Type Culture Collection (ATCC, Manassas, VA, USA) and cultured in Minimum Essential Medium (MEM) (Gibco, #10370) supplemented with 10% Fetal Bovine Serum (Gibco, #10437) and 1% P/S (Gibco, #15140). HepG2 cells were transfected using the *trans*IT-LT1 transfection reagent (Mirus, #MIR 2300). 3T3-L1 preadipocytes were maintained at 37°C, 5*%* CO_2_ in Dulbecco's Modified Eagle Medium (Gibco, #11965) supplemented with 10% Calf Serum (JRH, #12138C) and 1% P/S (Gibco, #15140).

### Protocol for MDH staining of LDs in living cells

To stain LDs in live cells with MDH (Abgent, #SM1000a), cells need to be first washed once with PBS, and incubated with 100 µM of MDH-containing PBS (37°C, 15 minutes). Cells should then be washed three times with PBS and transferred into phenol-red free medium for imaging. LDs can then be imaged using 405 nm excitation and 420–480 nm emission.

### 3T3-L1 Differentiation

In adipocyte differentiation, the preadipocytes were grown to confluency in 10% calf serum/DMEM. Two days (DAY 0) post confluency, the maintaining medium was switched to MDI induction medium (10% FBS/DMEM supplemented with 1.15×10^−4^ g/ml IBMX, 1 µg/ml Insulin and 1 µM Dexamethasone). The medium was further changed to insulin medium (10% FBS/DMEM with 1 µg/ml Insulin) on DAY 2, and returned to 10% FBS/DMEM from DAY 4 onwards.

### Fluorescence Spectroscopy

The MDH fluorescent emission spectrum in H_2_O, methanol, isopropanol, and sunflower seed oil were recorded on a Fluoromax-4 spectrofluorometer. MDH was excited with 405 nm, and its fluorescence between 420–670 nm was measured.

### Live Cell Imaging

Cells were imaged on an Olympus FV1000 confocal microscope under 5% CO_2_ at 37°C. MDH was excited with 405 nm, and its fluorescence between 420–480 nm was collected. BODIPY 493/503 (stained with 1 µg/ml in PBS for 15 min at room temperature; stocked at 1 mg/ml in methanol), was excited with 488 nm, and its fluorescence between 500–550 nm was collected. NileRed (stained with 100 ng/ml in PBS for 15 min at room temperature; stocked at 25 µg/ml in DMSO), was excited with 488 nm, and its fluorescence between 530–600 nm was collected. EGFP and mEos2 fluorescence were collected using 488 nm excitation (collecting 500–550 nm emission), and TagRFP fluorescence was obtained using 559 nm excitation (collecting 570–670 nm emission).

#### Two-photon Fluorescence Imaging

Cells were imaged on a Zeiss LSM 510 confocal microscope under 5% CO_2_ at 37°C. Images were collected using a 40×1.2 W Zeiss C-Apochromat objective. MDH was excited with 760 nm light (Coherent Chameleon), and its fluorescence between 435–485 nm was collected.

### Tracking LDs

HeLa cells stained with MDH or BODIPY 493/503 were imaged at 2-second time intervals for a total of 40 seconds, and the movements of LDs were tracked using the ImageJ particle tracker plugin. LDs trajectories that displayed continuous active transport throughout the entire movie were collected and analyzed.

## Results

### MDH solvatochromatic fluorescence properties

**Figure 1:**
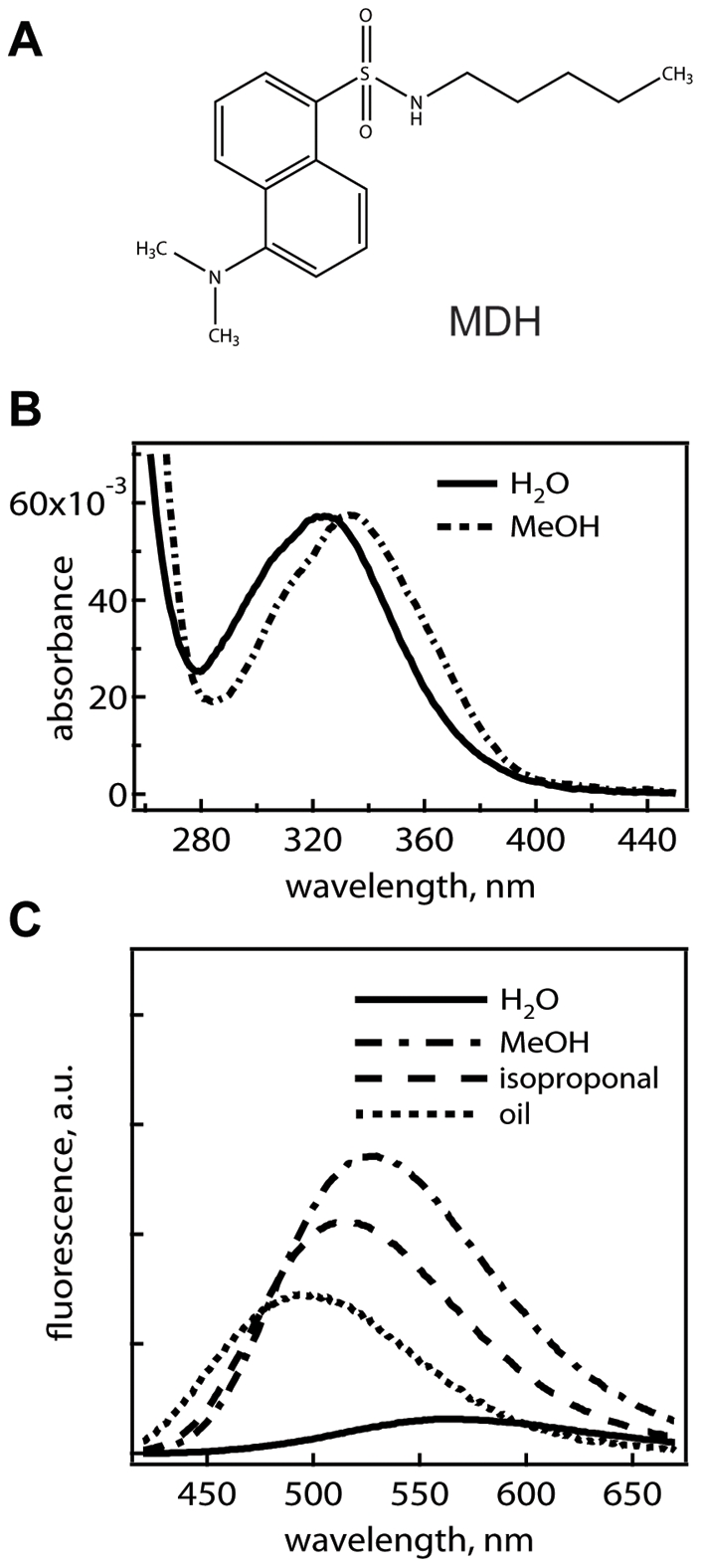
The Solvatochromatic properties of MDH. (A) MDH contains a fluorescent dansyl moiety that displays strong solvatochromatic properties. (B) MDH absorption spectrum in water and methanol. (C) MDH emission in different solvents (H_2_O/methanol/isopropanol/sunflower seed oil); the MDH emission maxima shifted from 570 nm to 485 nm with decreasing solvent polarity (when excited with 405 nm).

MDH contains a dansyl moiety known to exhibit solvatochromatic fluorescence properties [22], [23] (figure 1A); such properties provide microenvironment sensing ability that generates contrast for cell imaging. MDH absorbs in the violet, very well-separated from the most robust genetically-encoded fluorophores (figure 1B), the green and red-colored fluorescent reporters (which absorbs in the blue/green region of the visible spectrum), allowing them to be combined for multi-color live cell imaging. We find when using 405 nm excitation, a light source commonly found in confocal fluorescence microscopes, MDH's fluorescence displayed a strong polarity dependence. Not only did the emission intensity increase strongly in the non-polar solvents (i.e. methanol or isopropanol vs. water), the emission maximum was also greatly blue-shifted from 570 nm in water to 485 nm in sunflower seed oil (figure 1C). The effect was not a result of an MDH absorption difference in varying solvents (figure 1).

### Blue-fluorescing MDH's selectively labels LDs

**Figure 2:**
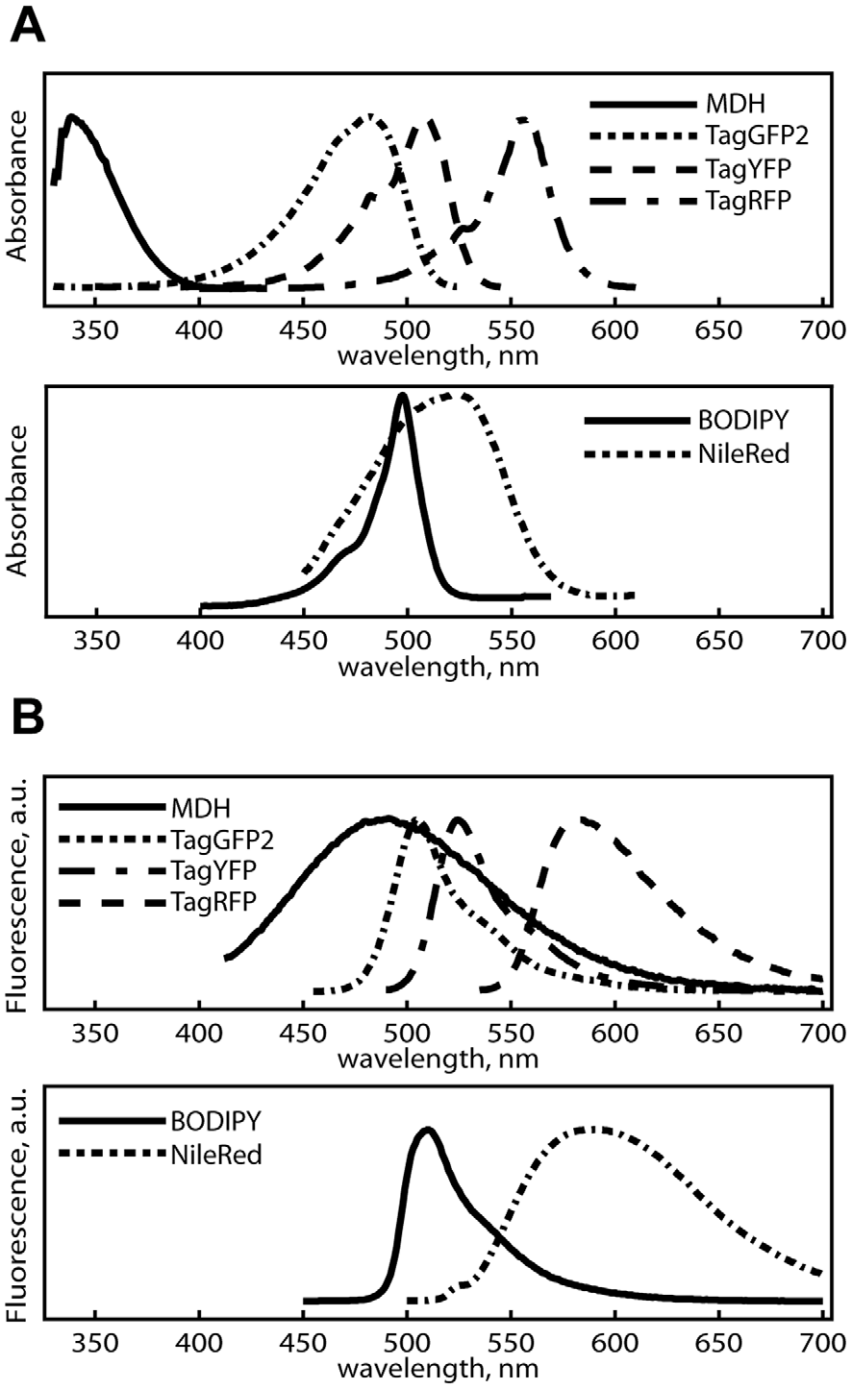
A comparison between the spectral properties of commercially available LD dyes and fluorescent proteins. The absorbance (A) and emission spectra (B) of LD dyes in sunflower seed oil, overlaid with fluorescent proteins' respective curves in water (the TagGFP2, TagYFP, and TagRFP spectra were obtained from Evrogen). The absorbance spectrum of MDH is significantly blue-shifted compared to those of the green and red fluorescent proteins. The 420–480 nm emission from MDH in oil is also free of overlap from the green and red fluorophores.

As MDH is hydrophobic, together with the fact that it emits strong blue fluorescence when placed into lipophilic environments (i.e. sunflower seed oil, figure 1), we reasoned that MDH could be a robust marker for LDs, the most hydrophobic structure of a cell. Cells labeled with MDH could be monitored for blue emission (420–480 nm, a spectral region free of potential background from the MDH that partitioned into aqueous environments) following 405 nm excitation to highlight cytosolic LDs. We compared the absorbance and emission spectrum of MDH and the commercially available LDs dyes in sunflower seed oil (to mimic the LD environment) to those of fluorescent proteins in water; MDH's absorbance and emission is significantly blue-shifted from green/red fluorescent proteins (figure 2). The 420–480 nm emission window is particularly suitable for imaging MDH within lipidic environments, and can be used in combination with green/red fluorophores for multicolor imaging.

**Figure 3:**
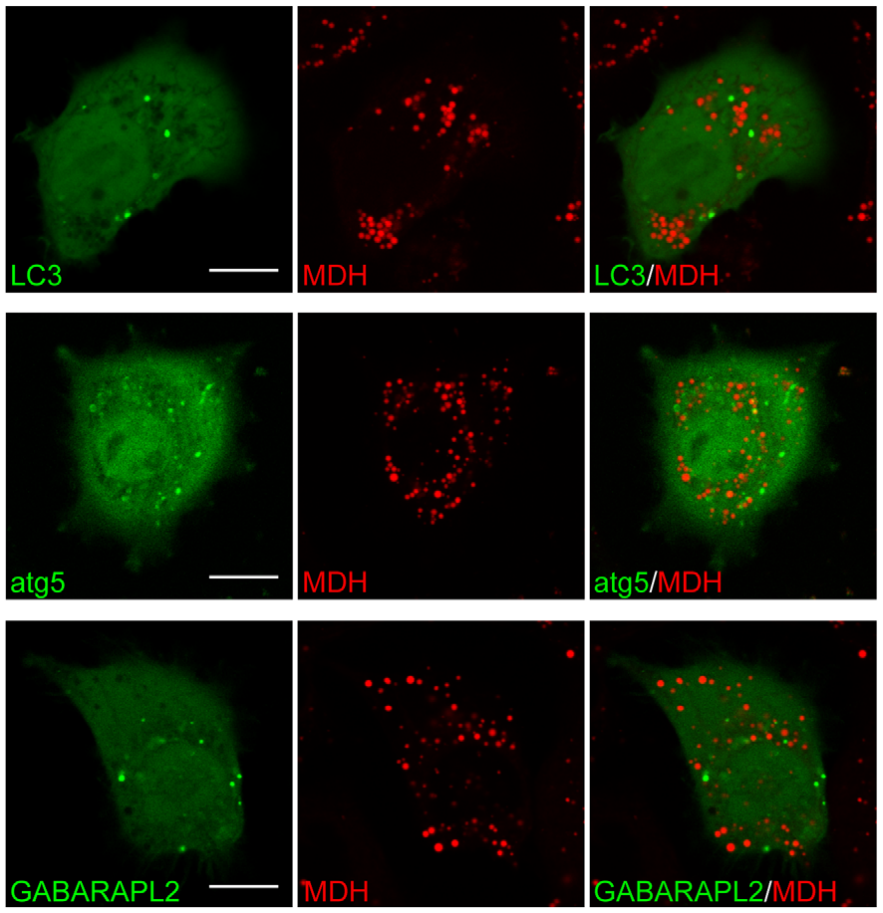
MDH staining patterns are separated from known molecular markers for autophagic structures. HepG2 cells transiently transfected with either EGFP-LC3B (*top row*), mcherry-atg5 (*middle row*), or mEos2-GABARAPL2 (*bottom row*) were stained with MDH and imaged. Under normal (not shown) and starvation conditions, EGFP-LC3B, TagRFP-atg5, or mEos2-GABARAPL2 puncta did not colocalize with MDH spots (*right columns; merged*). Scale bars, 10 µm.

MDH was previously proposed to be a marker for autophagic structures, due to its structural homology to a commonly utilized autophagosome dye, monodansylcadaverine (MDC) [24], [25]. However, MDH is much more hydrophobic than MDC, and chemical structures similar to MDH such as Prodan and Laurdan are known to partition into lipidic environments [26], [27]. In the initial report that suggested the use of MDH for autophagosome labeling, the signals were not verified with autophagic structure marker proteins, and were widefield fluorescence images on fixed cells showing strong cytoplasmic labeling that are difficult to interpret [25]. We verified the cellular staining patterns for MDH using live HepG2 cells, and found that MDH staining patterns (using 420–480 nm emission) were entirely segregated from well-known autophagic structure markers: this includes atg5, which resides on phagophores [28], and LC3/GABARAPL2, two molecules that resides on autophagosomal membranes [29] (figure 3). MDH structures also did not colocalize with LC3 in HeLa cells (figure S1). Instead, MDH staining patterns were apparently highly circular in shape, resembling cytosolic LDs.

**Figure 4:**
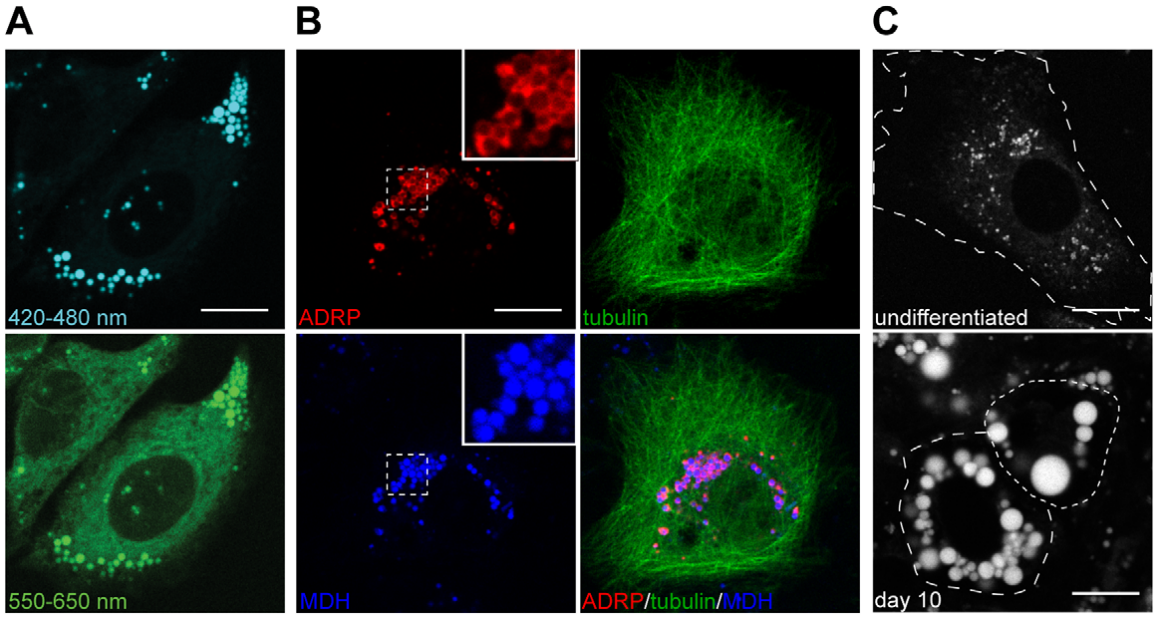
MDH stains LDs. (A) MDH staining patterns in HepG2 cells mimicked cytosolic LDs. Fluorescence emission between 420–480 nm resulted in a high contrast, low background staining of round spots in HepG2 (*top*; 405 nm excitation). In contrast, emission between 550–650 nm generated images retaining the round spots, but with diffusive background stains (*bottom*). (B) HepG2 cells were transiently cotransfected with TagRFP-ADRP (*colored red*) and EGFP-tubulin (*colored green*), and stained with MDH (*colored blue*). ADRP signals surrounded the MDH spots (Inset; magnified image of the white dotted square region), indicating that MDH patterns are indeed LDs. MDH can be combined with the use of green and red fluorophores, as evidence by the separate MDH/ADRP/tubulin images. (c) MDH specifically stained the LDs in 3T3-L1 cells before and after differentiation. *Top:* undifferentiated. *Bottom:* ten days after differentiation, showing the expected increase in average LD size. Scale bars, 10 µm.

To confirm that MDH staining patterns indeed represented LDs, we compared it to the cellular distribution of adipocyte-differentiation related protein (ADRP), an abundant LD surface protein [30]. We found that TagRFP-ADRP quantitatively decorated the surface of MDH stained puncta (full overlap), indicating that the MDH structures are cytosolic LDs (figure 4B). In addition, EGFP-TIP47 translocated onto MDH stained puncta upon oleic acid supplementation, again confirming MDH structures as LDs (figure S2). We further tested MDH's ability to stain LD in adipocytes, where neutral lipid accumulation is most abundant. As expected, before 3T3-L1s were differentiated (preadipocytes), the MDH patterns highlighted the small and dispersed LDs within the cell cytoplasm; 10 days after differentiation, the MDH patterns revealed large LDs, characteristic of the adipocyte phenotype (figure 4C).

### Mechanism for MDH-based LD imaging

By exciting MDH with 405 nm, and simultaneously imaging its fluorescence between 420–480 nm and 550–650 nm in HepG2 cells, we found that MDH's robust ability in highlighting cytosolic LDs can be attributed to two major factors: 1. MDH is hydrophobic and preferentially partitions into LDs. As shown in the fluorescence emission spectrum of MDH in figure 1 (*bottom*), MDH possess similar total fluorescence in water as in sunflower seed oil between 550–650 nm when excited with 405 nm. In cell images on MDH taken between 550–650 nm, LDs showed the strongest fluorescence (figure 4A, *bottom*, LDs were much brighter than other signals), indicating a preferential MDH-partitioning into LDs. 2: MDH fluorescence is highly enhanced and blue-shifted in lipophilic environments. While MDH preferentially partitions into lipophilic environments, imaging between 550–650 nm was not sufficient to generate a background-free image on cellular LDs. Collecting MDH's fluorescence between 420–480 nm, on the other hand, generated LD images devoid of background (figure 4A, *top*). This is because MDH's blue fluorescence is highly enhanced within nonpolar environments, while MDH in aqueous environments (i.e. figure 1, water) show no prominent signal within this emission range.

### Multi-color Live cell imaging using MDH

MDH's violet absorbance and its blue emission in lipophilic environments allow simple implementation of multicolor live-cell imaging on LDs. As shown in figure 4B, we simultaneously imaged LD with MDH (blue), the microtubule cytoskeleton with EGFP-tubulin (green, the track LDs travel on), and the ubiquitous LD protein TagRFP-ADRP (red). The images show no crosstalk between the 3 colors (a 2-color MDH/EGFP-tubulin movie is shown in Movie S1). It will also be straightforward to combine the use of MDH with other fluorescence reporters.

**Figure 5:**
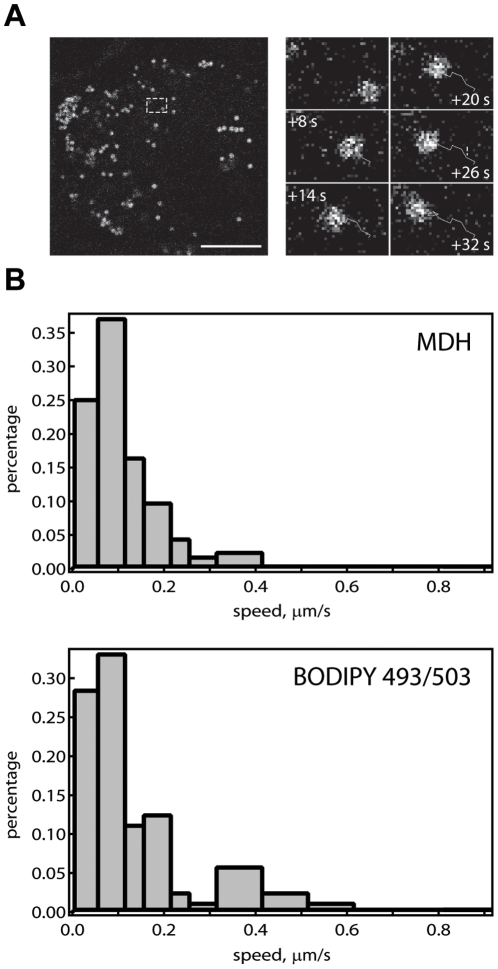
LD trafficking monitored in living cells using MDH. HeLa cells stained with MDH (A, *left*) were imaged every 2 seconds for 20 frames. Single LD trajectories (A, *right*, showing the movement of the LD in the white dotted rectangle) were then individually analyzed. LDs stained by MDH (B, *top*) showed similar active transport speeds compared to those stained with BODIPY 493/503 (B, *bottom*) (150 velocities each, in HeLa cells). The two distributions displayed similar mean (0.11 µm/s vs. 0.12) and standard deviation (0.09 vs. 0.11).

It is important to determine whether MDH staining retain the native cellular LD properties to allow imaging in living cells. We addressed this by monitoring LDs movements (motor protein driven active transport, figure 5A and Movie S2) on microtublues. We compared the MDH- and BODIPY 493/503- stained LD motilities in HeLa cells. The LD velocity distributions obtained using the two different dyes were apparently identical (figure 5B). The numbers matched the velocities of microtubule motor proteins [31], [32], and the LD movement speed previously obtained [33], [34]. This confirms that MDH is indeed compatible with live cell imaging of LD activities.

**Figure 6:**
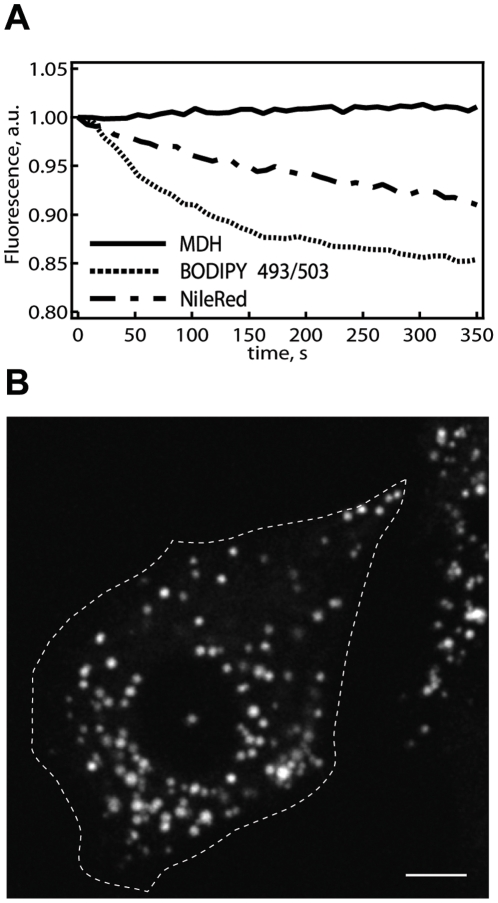
Superior MDH properties for live cell imaging. (A) The performance of MDH for long-term imaging was benchmarked against that of NileRed and BODIPY 493/503 directly in living cells. We tracked the fluorescence from MDH, NileRed, or BODIPY 493/503 stained, large/immobile LDs in differentiated 3T3-L1 cells over time (excitation laser powers were adjusted such that the images from the three respective dyes were of the same quality; Figure S3), and found that while NileRed and BODIPY 493/503 showed substantial photobleaching, MDH emission remained stable for long periods of time. (B) The LDs of HepG2 cells stained with MDH can be imaged with high contrast through two-photon fluorescence microscopy (760 nm excitation). The white-dotted line outlines the boundary of the cell. Scale bar, 10 µm.

We further benchmarked the performance of MDH against BODIPY 493/503 and NileRed for long term live-cell imaging. To perform this comparison, we stained differentiated 3T3-L1 adipocytes with MDH, BODIPY 493/503, or NileRed. The differentiated cells display large (>5 µm) LDs that remains immobile, thereby trackable and permits quantification of the fluorescence emission over time (which avoids the complication from stage drift and cell movement). Adjusting the respective excitation laser powers (405 nm for MDH, 488 nm for BODIPY 493/503 and NileRed) so that images from the two dyes are of the same quality (figure S3), we then continuously imaged the LDs for over 300 seconds. In contrast to BODIPY 493/503 and NileRed, which showed marked photobleaching, MDH fluorescence in LDs remained unchanged (figure 6A), demonstrating its superior performance for live cell imaging.

In addition to its use in living cells, MDH can potentially be extended towards the imaging of LD in intact organisms, such as in *C. elegans*. Fluorescence imaging in organisms oftentimes mandates the use two-photon excitation (TPE), and we found that MDH is compatible with such configurations. As shown in figure 6B, MDH stained HepG2 cells can be effectively imaged with TPE microscopy while again maintaining high photostability.

## Discussion

Here we have demonstrated that the commercially available monodansylpentane serves as a robust marker for LDs in living cells. Its absorption in the violet region and the blue-fluorescence allow MDH to be separated with the most powerful fluorescence reporters. We found that MDH achieved its exquisite labeling on LD through two properties: its lipophilic nature and its solvatochromatic fluorescence properties. MDH is highly photostable, and can be efficiently excited using 405 nm laser light commonly available on confocal fluorescence microscopes. MDH staining with the supplied protocol allows long term live-cell imaging, without apparent effects to the cells. Taken together, MDH should represent an attractive option for researchers wanting to image dynamic regulation of LD in living cells using fluorescence microscopy.

## Supporting Information

Figure S1 **Lack of colocalization between EGFP-LC3 and MDH-stained puncta in starved (EBSS, 1 hour) HeLa cells.** Scale bar, 10 µm.
(TIF)

Figure S2 **Translocation of Tip47 onto MDH puncta upon oleic acid supplementation.** In HepG2 cells, EGFP-TIP47 resided in the cytosol when cultured in MEM+10% FBS (**A**), but translocated onto MDH stained puncta upon eight hours of oleic acid supplementation (0.4 mM oleic acid complexed with 0.25 mM BSA in MEM/FBS, **B**). Scale bars, 10 µm.
(TIF)

Figure S3 **Image quality adjustment for LD dye comparison.** Using the same detector (PMT) and scan settings, we varied the respective laser excitation powers to achieve similar image quality for MDH-, BODIPY 493/503-, and NileRed-stained LD images in differentiated 3T3-L1 cells (8 days) for performance comparison. For example, MDH stained 3T3-L1 cells were imaged at various excitation powers (in this figure 1× represents the final excitation power we chose for MDH imaging; 0.5× = 50% of the laser intensity used in our performance test; 2× = 200% of the laser intensity used in the performance test), and the emission intensity profile for large (>5 µm), immobile LDs were analyzed (*right*, cross-section of the white bar indicated in the 0.5× image). The excitation powers that produced an S/N = 5 on these large LDs for the respective dyes were selected as the laser intensity to use for further performance evaluation.
(TIF)

Movie S1 **2-color live-cell imaging with MDH in HepG2 cells.** HepG2 cells transfected with EGFP-tubulin (shown in green) were stained with MDH (shown in cyan) and imaged every 30 seconds for 15 frames.
(AVI)

Movie S2 **LD active transport in HeLa cells.** HeLa cells was imaged every 0.14 seconds for 20 frames and analyzed for LD trajectories (corresponding movie file for figure 5).
(AVI)
