# Supplementary figures and images for: Monodansylpentane as a Blue-Fluorescent Lipid-Droplet Marker for Multi-Color Live-Cell Imaging
Source: PLoS One. 2012 Mar 1;7(3):e32693. doi: 10.1371/journal.pone.0032693 (PMC3291611; doi:10.1371/journal.pone.0032693)

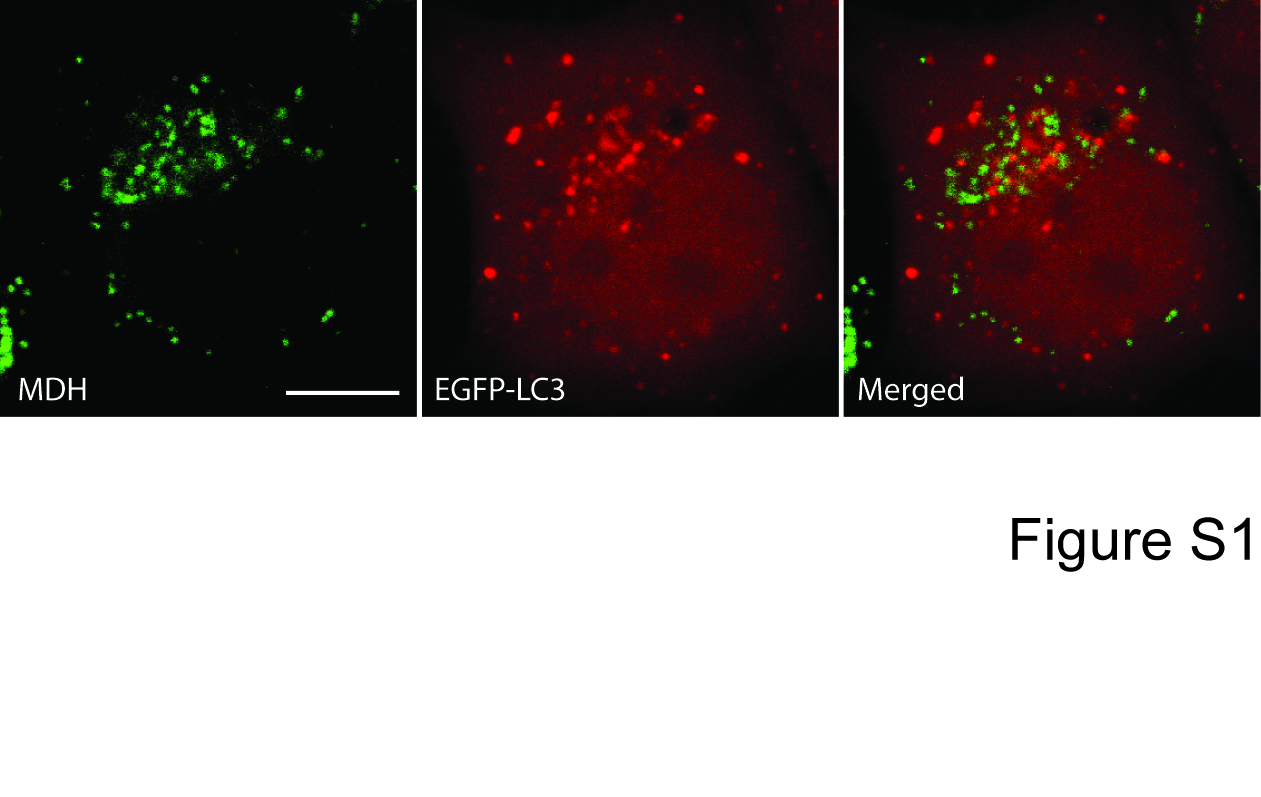

Supplement: Figure S1 — Lack of colocalization between EGFP-LC3 and MDH-stained puncta in starved (EBSS, 1 hour) HeLa cells. Scale bar, 10 µm. (TIF) [file pone.0032693.s001.tif]

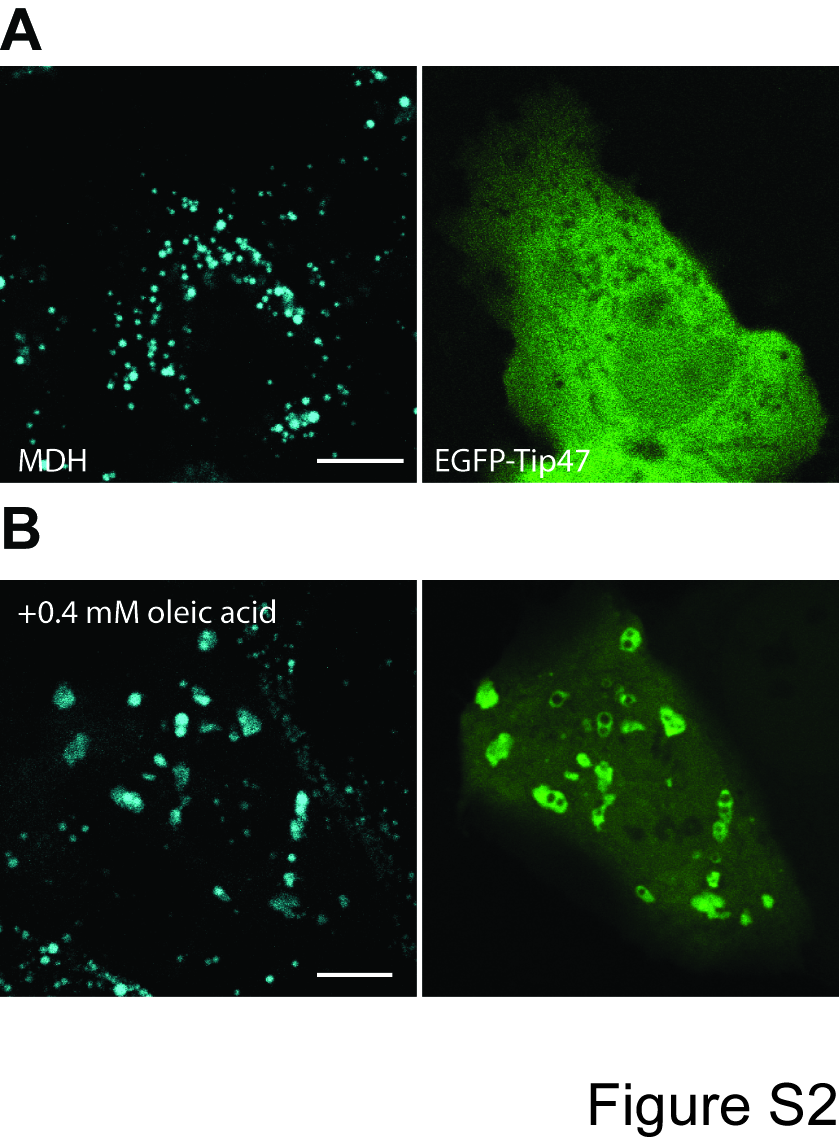

Supplement: Figure S2 — Translocation of Tip47 onto MDH puncta upon oleic acid supplementation. In HepG2 cells, EGFP-TIP47 resided in the cytosol when cultured in MEM+10% FBS (A), but translocated onto MDH stained puncta upon eight hours of oleic acid supplementation (0.4 mM oleic acid complexed with 0.25 mM BSA in MEM/FBS, B). Scale bars, 10 µm. (TIF) [file pone.0032693.s002.tif]

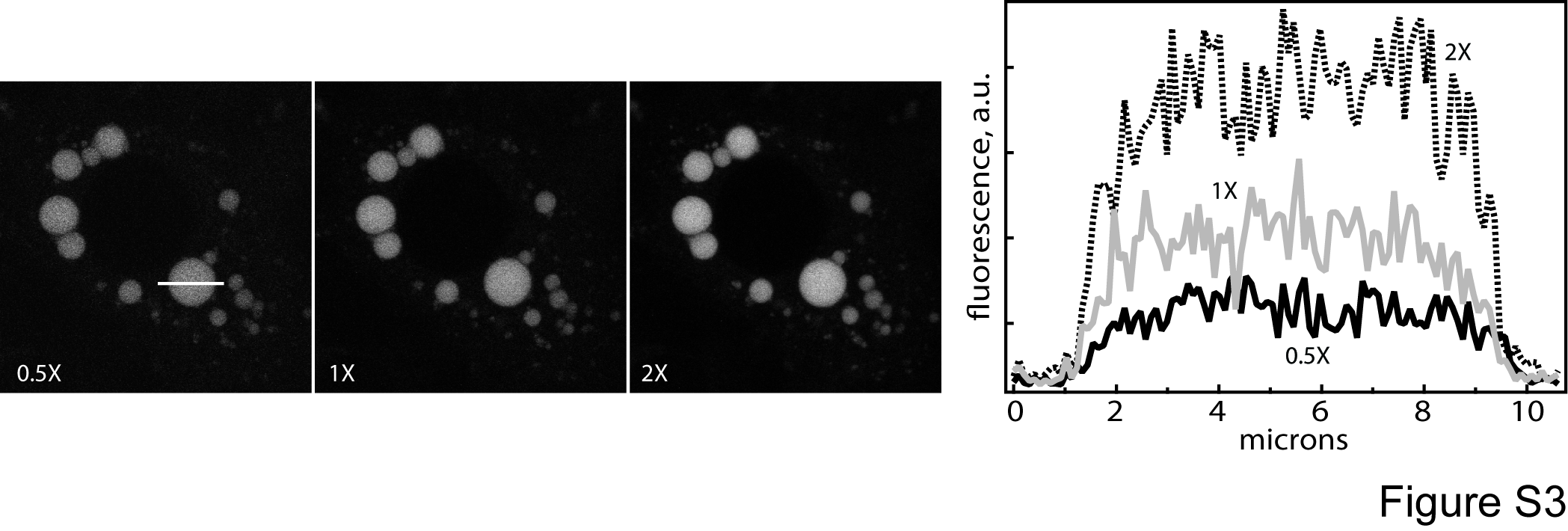

Supplement: Figure S3 — Image quality adjustment for LD dye comparison. Using the same detector (PMT) and scan settings, we varied the respective laser excitation powers to achieve similar image quality for MDH-, BODIPY 493/503-, and NileRed-stained LD images in differentiated 3T3-L1 cells (8 days) for performance comparison. For example, MDH stained 3T3-L1 cells were imaged at various excitation powers (in this figure 1× represents the final excitation power we chose for MDH imaging; 0.5× = 50% of the laser intensity used in our performance test; 2× = 200% of the laser intensity used in the performance test), and the emission intensity profile for large (>5 µm), immobile LDs were analyzed (right, cross-section of the white bar indicated in the 0.5× image). The excitation powers that produced an S/N = 5 on these large LDs for the respective dyes were selected as the laser intensity to use for further performance evaluation. (TIF) [file pone.0032693.s003.tif]
